# Spontaneous perforation of jejunal gastrintestinal stromal tumour (gist). Case report and review of literature

**DOI:** 10.1186/1749-7922-7-37

**Published:** 2012-11-29

**Authors:** Somsubhra Datta Roy, Dawood Khan, Krishna K De, Utpal De

**Affiliations:** 1 Department of surgery, Medical College Hospital, 88, College Street, Kolkata, 73, India; 2 Dankuni Housing Complex, L-4/9, Ph 3, Dankuni, Hooghly, 713211, India

**Keywords:** Jejunum, GIST, Perforative peritonitis

## Abstract

GIST is the commonest mesenchymal tumour of the gastrointestinal tract. Jejunal GIST is rare and spontaneous perforation of asymptomatic jejunal GIST is unique. A review of the English literature reveals only fifteen cases of perforated GIST till date. Pre-operative diagnosis is difficult. Diagnosis is confirmed on histopathology and immuno-histochemistry. Complete removal with postoperative imatinib therapy entails optimal treatment. Perforated GIST is associated with high recurrence.

## Background

Gastro-intestinal stromal tumour (GIST) is most common mesenchymal tumour of gastrointestinal tract (G.I) tract (80%) [1]. The incidence of GIST is 10–20 million people per year with a malignant potential of 20-30% [1,2]. Presentations include abdominal mass (5-50%), obstruction (5%), haemorrhage and rarely perforation (0.8%) [1,2]. Spontaneous perforation of jejunal GIST is rare (Table 1) and unique. This article is an illustration of a similar case.

**Table 1:** Table showing published case reports on jejunal perforation

**Serial number**	**Country**	**Journal**	**Patient paticulars**	**Date of publication**
1	Greece	Journal of gastro-intetinal and liver diseases	66 yrs/ M	2006
2 ^*^	China	Journal of Chinese oncology	69 yrs/ M	2008
3	Ankara	Turkish journal of gastroenterology	70 yrs/ M	2008
4	Turkey	Gastroenterology research	52 yrs / F	2009
5	Japan	Journal of abdominal emergency medicine	56 yrs/ M	2009
6 ^*^	China	International journal of gastroentrology	69 yrs/ M	2009
7	Greece	World journal of surgical oncology	28 yrs/ F	2010
8	Istanbul	Turkish journal of gastroenterology	65 yrs/ M	2010
9	India	International journal of biomedical research	68 yrs / M	2010
10	India	Bombay hospital journal	55 yrs/ M	2011
11	China	Turkish journal of gastroenterology	45 yrs/ M	2011
12	Greece	journal of current surgery	56 yrs/ M	2011
13	Turkey	Journal of clinical and analytical medicne	61 yrs/ F	2012
14	India	The internet journal of surgery	35 yrs/ M	2012
15	India	Indian journal of surgery	22 yrs/ M	2012

* same case report published in different journal.

## Case presentation

Forty six year old male presented to the emergency department with pain epigastrium, constipation, nausea, vomiting, fever and abdominal distension since two days. He had a past history of acid peptic disorder for which he was treated conservatively. On physical examination, patient was conscious and of normal built. Pallor, cyanosis, icterus and edema were absent. He was normotensive (124/70 mmHg), had tachycardia (110/min), fever (102.4°F) and hurried respiration (25/min). Abdominal examination revealed distension, board like rigidity, marked rebound tenderness, absent liver dullness and inaudible bowel sounds. Hernia sites were normal. Per-rectal examination did not reveal any significant abnormality. Examinations of other systems were within normal limits. A provisional diagnosis of peptic perforation was made.

Exploratory laparotomy was planned. Hematological examination revealed mild anemic with neutophilic leucocytosis [Hemoglobin – 9.8 g/dl, Total count- 14,000/cu.mm (N85, L11, E10, B0, M0)]. Blood sugar (113 g/dl), liver function tests and serum electrolytes (Na-136 meq/lit, K- 4.2 meq/lit) were within normal limits. Viral markers were non-reactive. Abdominal roentgenogram showed free gas under both domes of diaphragm with diffuse ground glass opacity. Excessive gas in the abdomen with free fluid was noted in abdominal sonography.

**Figure 1:**
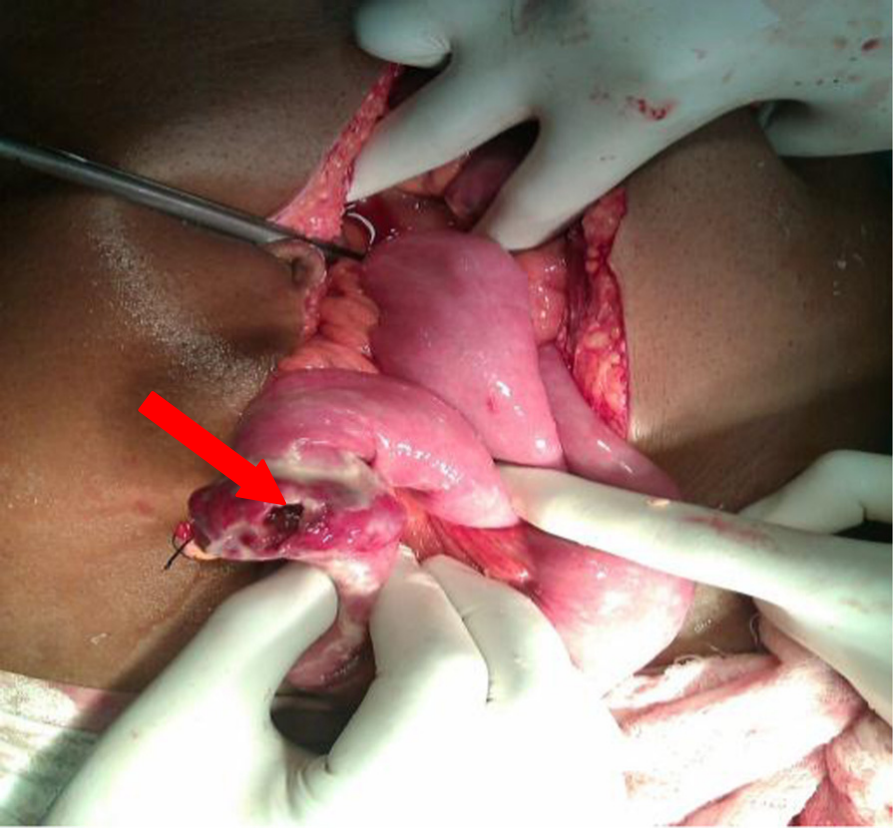
Peroperative photograph showing jejunal gist with perforation.

The patient was resuscitated with intravenous fluids, ryles tube and antibiotics. Following adequate resuscitation, the patient was put up for operation. Midline laparotomy revealed purulent free fluid with flakes. On aspiration and removal of the flakes and fluid, a purplish coloured firm growth with everted margins, measuring 3×2 cm was found in the anti-mesenteric border of the jejunum, fifty cm from the duodeno-jejunal flexure. The growth had a central perforation with intestinal contents effusing through the rent (Figure 1). All other organs were normal. The growth was resected with five cm margin and an end to end, single layer, interrupted, anastomosis was performed using 2^′^0^′^ polyglycolic suture. Thorough peritoneal lavage was done with warm normal saline and abdomen was closed in layers. A tube drain was placed in the hepatorenal pouch of Morrison. The specimen was sent for histopathological examination.

Post operative period was uneventful and the patient was discharged on the tenth post-op day after stitch removal.

**Figure 2:**
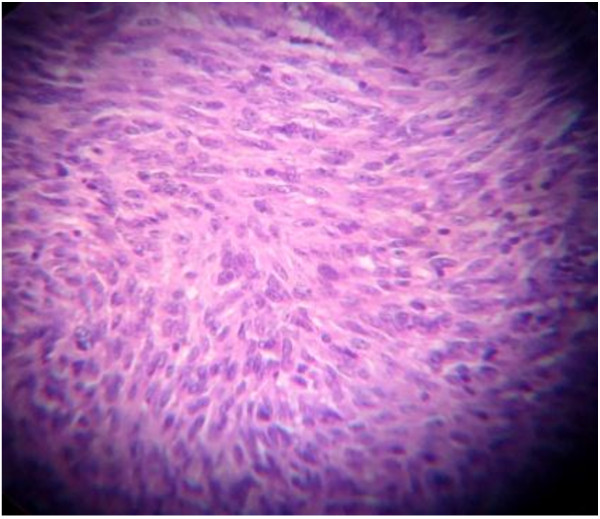
Histopathology of jejunal GIST.

**Figure 3:**
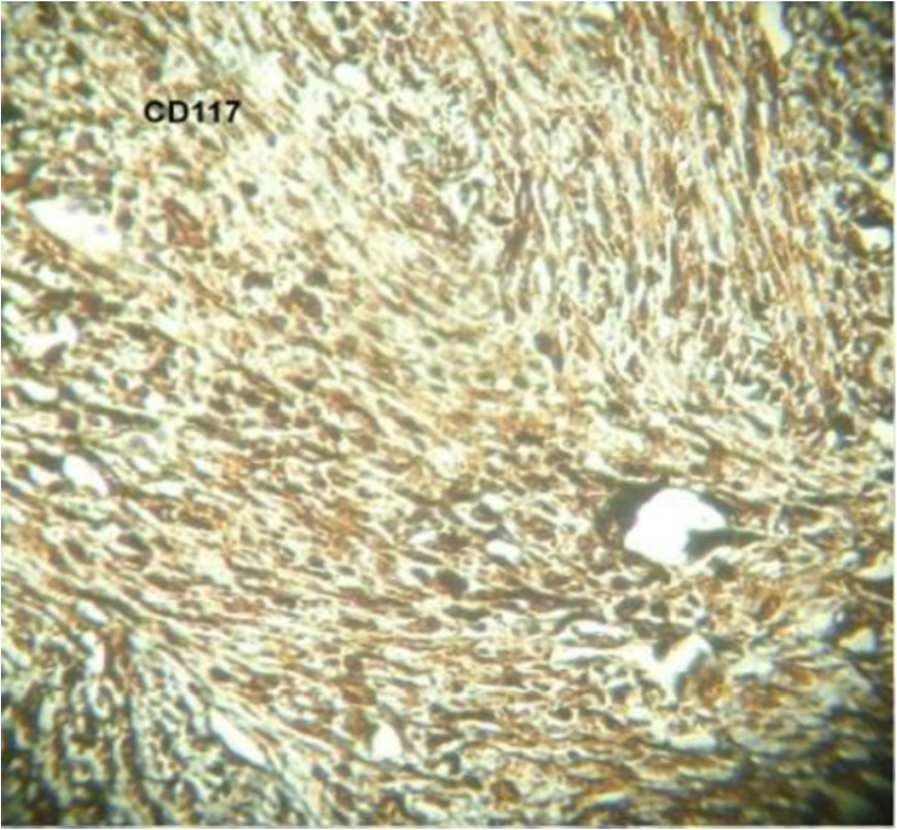
Immunohistochemistry for CD117 positivity.

Histopathology (Figure 2) of the resected specimen showed, a submucosal nodular tumour composed of interlacing fascicles of spindle shaped cells with elongated, plump nuclei. There was mild nuclear pleomorphism and more than five mitotic figures per fifty high power fields. No tumour necrosis found. Pathologically it was jejunal GIST of intermediate risk. Surgical lines of resection were free. Immuno-histochemistry study revealed diffuse immunoreactivity for CD-117 (Figure 3), focal CD-34 positivity, negative for desmin, S-100 and SMA;Ki 67 less than 5%.

The patient is on six months follow-up receiving oral imatinib 300 mg twice a day.

## Conclusion

GIST was first described by Mazor and Clark (1983) [1]. It originates from the interstitial cells of Cajal (ICC), located in the muscularis propria (myenteric plexus) responsible for triggering smooth muscle contraction [2,3]. The basic pathology is an activating mutation (gain in function) of chromosome 4 which codes for c-Kit resulting in uncontrolled proliferation of stem cells that differentiate towards ICC. GIST is sporadic [3]. Familial forms with autosomal dominant inheritance have also been documented [3,4].

Isolated reports of GIST occurring concomitantly with paraganglioma, pulmonary chondroma, nerofibromatosis, pancreatic neuro-endocrine tumours, burkitt’s lymphoma, osteosarcoma, neuroblastoma and melanoma have been documented [4].

90% of GIST occurs in adults more than 40 years of age (median age 63 years). There is slight male preponderance [4]. No documented elements indicating any association with geographic location, ethnicity, race or occupation has been elucidated [4,5].

The commonest site of GIST is stomach (60-70%) [2,3]. Jejunum accounts for 10% of all GI tract GIST’s [1,3]. Sporadic reports of GISTs arising from the omentum, mesentery or retroperitoneum, have been documented but most of these are metastatic from gastric or intestinal primaries [4]. Extra-GIST has been reported in gall bladder, pancreas, liver and urinary bladder [4].

Presentation is erratic. Seventy percent are symptomatic at presentation, 20% are asymptomatic and 10% are detected at autopsy [5,6]. Common presentations include abdominal pain, palpable mass, gastro intestinal bleeding, fever, anorexia, weight loss and anaemia [7]. Isolated jejunal GIST associated with perforation and peritonitis is a rare and unique [1].

Perforation is usually attributed to replacement of bowel wall by tumour cells, tumour embolization leading to ischemia, necrosis together with raised intra-luminal pressure [4,5,7]. In view of the exophytic nature of the growth, intestinal obstruction occurs due to compression rather than luminal obstruction. As such intetstinal obstruction is a rare occurrence until the tumour attains enormous size.

Clinical diagnosis of GIST is based on index of suspicion [6,7]. Specific diagnostic signs and symptoms are absent. Chronicity is a rule. Acute atypical presentation includes hemorrhage and perforative peritonitis [1-10]. Preoperative imaging modalities like contrast enhanced abdominal computerized tomography (CT) aids in diagnosis [8]. The extent of the tumor, metastases and involvement of other organs can be assessed. A dedicated magnetic resonance imaging (MRI) provides better information than CT in the preoperative staging workup [7,8]. Endoscopy can diagnose gastric GISTs. Endoscopy demonstrates smooth, mucosa-lined protrusion of the bowel wall which may or may not show signs of bleeding or ulceration. Endoscopic ultrasound with guided fine-needle aspiration is diagnostic for primary lesions in 89% of the cases [8-10]. However, the relative usefulness of images depends on the site, duration and suspicion of GIST in patients presenting with undiagnosed abdominal lumps. The decisive diagnosis rests on the pathological and immunohistological tests [2,5-10]. Histopathologically GISTs are composed of spindle (70%), epithelioid and round cell or an admixture [6,8]. Similarities with histological picture of gastrointestinal leiomyosarcoma, leiomyoblastoma and poorly differentiated carcinomas may cause diagnostic dielemma, Immuno-histochemical assays for CD117 antigen (KIT) is the mainstay for diagnosis [9,10].

Diagnosis of asymptomatic GIST with acute presentation like perforation remains elusive. Accordingly, our provisional diagnosis was peptic perforation as free gas under diaphragm was noted in erect abdominal rhoentgenogram.

Optimal surgical treatment of GIST entails complete removal of the tumor with clear surgical margins including the adjacent involved organs [5-10]. Complete surgical resection entails 48-65% five-year survival [1]. Perforation of the tumor lowers the five-year survival to 24%, probably due to peritoneal dissemination [5]. Local and regional lymph node involvement is infrequent in GIST [6,8,10]. GIST’s presenting with perforation, attention needs to be paid, in view of possible recurrence of the tumor. Abundant peritoneal lavage should be performed with distilled water to reduce the risk of peritoneal tumour spillage. Distilled water is used because of its cytolytic activity on suspended cells [7,9,10].

GIST response to conventional chemotherapy is very poor (<10%), while radiotherapy is only used in cases of intraperitoneal hemorrhage, when the precise location of the tumor is known, or for analgesic purposes [7,8].

STI571 (imatinib), acts as a powerful selective inhibitor of tyrosine-kinase, PDGFR (platelet derived growth factor receptor) and c-kit receptor [10]. Oral imatinib at doses >300 mg per day achieves curative results.

The prognostic factors of GIST include age at presentation, anatomic location, size (most important), histomorphology, immuno-histochemistry and molecular genetics [4,6-10].

Positron-emission tomography with 18F-fluoro-2-deoxy-D-glucose is a very useful tool for the postoperative follow-up of patients receiving imatinib [4,5,9,10].

The 5-year survival rate is 35%. It increases to 54% after complete surgical excision [1-10]. However 40% will recur within 18 – 24 months. Once recurrence has occurred median survival is 9–16 months [3,5,7,8,10].

## Consent

“Written informed consent was obtained from the patient for publication of this Case report and any accompanying images. A copy of the written consent is available for review by the Editor-in-Chief of this journal”.

## Competing interests

The authors declare that they have no competing interests.

## Authors' contributions

UD participated in the conception, design of the study, sequence alignment and drafted the manuscript. SD carried out the immunohistochemical studies. DK participated in the clinical and surgical management. KKD helped to draft the manuscript. All authors read and approved the final manuscript.
